# Educational challenges as experienced by pregnant student nurses at a college in Mpumalanga

**DOI:** 10.4102/curationis.v42i1.1880

**Published:** 2019-06-06

**Authors:** Khothasane B. Nkosi, Agnes Makhene, Sidwell Matlala

**Affiliations:** 1 Mpumalanga Department of Health, Mpumalanga College of Nursing, Nelspruit, South Africa; 2 Department of Nursing Science, University of Johannesburg, Johannesburg, South Africa

**Keywords:** challenge, college, educational, pregnant, student

## Abstract

**Background:**

Student nurses at a college in Mpumalanga fall pregnant before they complete their training, and some commence training while pregnant and face educational challenges in both theoretical and clinical learning areas. It becomes impossible for them to complete their training on time.

**Objectives:**

The objectives of this study were to explore and describe educational challenges as experienced by pregnant student nurses at a college in Mpumalanga and to formulate recommendations that can be used by the college and pregnant student nurses to address their educational challenges.

**Methods:**

A qualitative, exploratory, descriptive and contextual research design was used. Ten student nurses were selected through purposive sampling. Data were collected by means of in-depth unstructured individual phenomenological interviews between September and November 2016. Data were analysed using Giorgi’s qualitative thematic analysis method.

**Results:**

The central theme that emerged from this study confirmed that pregnant student nurses experienced educational challenges negatively. Four main themes that emerged were academic challenges, failure to write examinations, support system and maternity leave. These findings had a negative impact on their education.

**Conclusion:**

There is a need for the college to minimise the identified educational challenges to promote completion of training on time.

## Introduction

In nursing colleges, pregnancies among student nurses remain a challenge. Apart from the social and psychological challenges, pregnancy can also affect the academic success of student nurses and that of the institutions (Coetzee & Ngunyulu 2015:1; Naidoo & Kasirum 2008:341; Van Den Berg & Mamhute 2013:305). Failure to write examinations and the lack of maternity benefits and support from colleagues and staff during pregnancy are among the challenges that pregnant student nurses face.

Karra and Lee (2012:4) point out that pregnant women may take longer to complete their studies because of the challenges they face, and this may impact the economy. In contrast, in the United Kingdom, pregnancy does not pose a challenge to student nurses as they receive support, are allowed to continue their studies and have 12 weeks of maternity leave as authorised absence (University of Salford 2015:6). However, they have to make up for the time lost to comply with the professional registration regulation, and to be able to write their examinations on their return (University of Salford 2015:7).

In Zimbabwe, pregnant student nurses may also take longer to complete their studies because of a policy that instructs school principals to exclude pregnant students who, because of pregnancy-related physical discomfort, are facing academic challenges (Van Den Berg & Mamhute 2013:305). At the KwaZulu-Natal College of Nursing (KZNCN), the introduction of a new bursary system meant that pregnant student nurses forfeited their maternity benefits for a period of 6 months as they were not officially allowed to fall pregnant during their training (Jacobs 2014:55).

Naidoo and Kasirum (2008:13) describe a similar situation and policies in Nigerian colleges and universities that allow for the expulsion of pregnant students. However, Mohale (2015:4) states that in a South African Military Nursing College (SAMNC), pregnant student nurses are allowed to continue with their training. However, from the majority of the sources, it seems that pregnant student nurses, in general, need more fair consideration and better support and guidance from the academic community and other stakeholders to successfully balance their role as mothers and students (Mamhute 2011:70).

Mpumalanga Province has one college that admits student nurses for the Diploma in Nursing (General, Community, Psychiatry) and Midwifery, in terms of South African Nursing Council (SANC) regulation R425 of 22 February 1985. The college has one intake per year between February and April. According to the college admission register, the intake comprises 70% females and 30% males, with candidates being between the ages of 18 and 35. Applicants from all racial groups who are South African citizens and live in the Mpumalanga Province are eligible for registration. Student nurses are given dual status as students and employees by the Mpumalanga Department of Health (MDOH). The admission criteria allow the college to cater for three municipal areas in Mpumalanga, with a population of approximately 4 million (Scheepers, Jacobs & Punt 2009:3).

The admission of pregnant student nurses to the course has brought some educational challenges. Finishing one’s training in the usual time has been impossible as pregnant student nurses miss some examinations because of pregnancy-related problems (Mamhute 2011:71). Information from the college archives shows that, in 2015, three student nurses were admitted while pregnant; seven in 2014; nine in 2013; 10 in 2012 and four in 2011. A total number of 34 student nurses were admitted at the college while pregnant between 2011 and 2015, excluding those who reported their pregnancy during the course of their studies. Without adequate support, these pregnant student nurses may struggle to complete their training (Sibeko 2012:9).

### Problem statement

South Africa actively promotes equity policies, and the admission of pregnant women in educational institutions and work environments forms part of these equity practices. Women are also supported by being granted maternity leave of not less than four consecutive months, according to the *Basic Conditions of Employment Act* No. 75 of 1997 (BCEA).

Sometimes, pregnant student nurses fail to write block tests that would qualify them for entrance into the examinations because they are not well, or they are already on maternity leave. Some of them hide their pregnancies for fear of being sent on maternity leave. Pregnant student nurses are conscious of the challenges of pregnancy to their academic performance (Kambanji 2010:2). For example, mental health institutions where they practise their psychiatric nursing component do not allow them to practise while they are pregnant.

Therefore, the problem statement is described as follows:

For the past five years, the college has encountered challenges as most student nurses were admitted to the college either while pregnant or they fell pregnant before they could finish their four years of training. (Sekgobela 2008:4; Zungu & Manyisa 2009:61)

*This has a negative influence on the nursing profession that is already burdened by a shortage of professional nurses.* This article seeks to address this problem. The research questions that emerged from the problem statement are as follows:

- What educational challenges are experienced by pregnant student nurses at a college in Mpumalanga?
- What recommendations can be made to the college and pregnant student nurses to address these educational challenges?

### Research objectives

The objectives of this study are the following:

- to explore and describe educational challenges as experienced by pregnant student nurses at a college in Mpumalanga
- to formulate recommendations that can be used by the college and pregnant student nurses to address their educational challenges.

## Research design and methods

### Research design

A qualitative, exploratory, descriptive and contextual research design was used (Grove, Burns & Gray 2013:195). A qualitative research method is largely an investigative process where the researcher makes sense of a phenomenon under study (Miles & Huberman 2009:104). An exploratory research design was followed to explore the lived experiences of pregnant student nurses. This research was also contextual as it was conducted at a college in Mpumalanga.

### Research methods

Phenomenological studies focus on exploring the lived experiences of individuals as they occur (Creswell 2014:14). Use of phenomenological method was facilitated through bracketing, to ensure neutrality and truth value of the findings without the researcher imposing preconceived ideas (Grove et al. 2013:60).

### Population and sample

Grove et al. (2013:44) define a population as the bigger pool from which sampling elements are drawn. In this study, the population is all female student nurses registered with the SANC for a Diploma in Nursing (general, psychiatry, community) and Midwifery at a college in Mpumalanga between 2013 and 2016.

A sample is a subset of population elements (Polit & Beck 2012:275). In the study, the sample includes student nurses admitted at the selected college while pregnant, those who became pregnant while studying and those who have already delivered their babies and are still at the college. The sampling method is the procedure of choosing a fraction of the population to represent the population being studied (Polit & Beck 2012:275). The sample size was determined by data saturation when additional sampling did not provide new information (Grove et al. 2013:371). In the study, non-probability purposive sampling was used, where the researcher consciously selected participants with rich information (Grove et al. 2013:365). The researcher requested student nurses who were pregnant and those who have given birth to be participants, after a thorough explanation of the study process and signing of informed consent.

### Data collection

Grove et al. (2013:45) define data collection as the exact, systematic gathering of information pertinent to the research purpose or the accurate objectives, questions or hypotheses of the study. Data were collected by means of unstructured individual phenomenological interviews that took place at a convenient venue free from noise and distractions and lasted about 30–40 min (Polit & Beck 2012:59). The researcher did not take part in the interview processes as she is known to the participants. A colleague with interviewing skills conducted the interviews. The interviewer asked for permission from participants to record all conversations on an audiotape.

The central questions posed during the interviews include the following:

- What were the educational challenges you experienced as a pregnant student nurse at this college?
- What do you recommend should be done to address these educational challenges?

### Data analysis

According to Grove et al. (2013:46), qualitative data analysis is done to reduce, sort and make sense of the data. This occurs after data collection (Polit & Beck 2012:60). Data were analysed according to Giorgi’s thematic data analysis method (Clarke & Braun 2013:4–5). The researcher identified the central theme, main themes and sub-themes. An independent coder verified the accuracy of the analysed data and had a consensus meeting with the researcher to discuss and agree upon the identified themes.

### Measures to ensure trustworthiness

Trustworthiness is the degree of confidence that the researcher has in the collected and analysed data, and the following was applied: credibility, transferability, confirmability and dependability (Lincoln & Guba 2005:290). To ensure credibility, the researcher and the independent interviewer established rapport and had extensive discussions with the participants. Thick descriptions of research methods and data also increased the credibility of the study (Lincoln & Guba 2005:290). Transferability was reached by using dense description of data and research methods in the research report and addition of verbatim quotes from participants to ensure precise description of the data (Polit & Beck 2012:585). Dependability was ensured by providing dense description of the research methodology, consultation from supervisors as experts in qualitative research throughout the study, as well as the use of an independent coder for consensus discussions on the themes and sub-themes that were identified. Confirmability was ensured by providing an audit trail as evidence of the research process (Polit & Beck 2012:495). An independent coder was asked to confirm the accuracy of the collected and analysed data.

### Ethical considerations

Ethical clearance was obtained from Mpumalanga Department of Health Ethics Committee (reference number: PHRCE MP_2016 RPO_562) as well as from the University of Johannesburg Ethics Committee (reference number: REC-01-121-2016). Ethical principles were complied with to protect the rights, dignity and safety of all participants (Grove et al. 2013:171). The researcher applied the following ethical principles, as outlined in Dhai and McQuoid-Mason (2011:14): respect for personal autonomy, the principle of beneficence and non-maleficence, informed consent, the principle of justice, and privacy and anonymity.

Consent was also given by the participants, and they were informed of their right to withdraw from the study at any time if they are no longer willing to participate (Creswell 2014:11). Code names were used to maintain anonymity (Grove et al. 2013:171).

## Discussion of findings

Following the unstructured individual interviews of participants, one central theme indicated that pregnant student nurses experienced educational challenges in a negative way. The following main themes emerged, namely academic challenges, failure to write examinations, support system and maternity leave. The descriptions of findings that were obtained from the interviews were integrated with the relevant literature to add to the body of knowledge. The description of themes is presented in the next section.

### Theme 1: Academic challenges

Participants reported lack of concentration, physical discomfort, failure to cope with workload and academic stress both in the classroom and at the clinical learning sites, which contributed to their academic challenges. This participant said:

‘You cannot easily concentrate, [*silence*]… you can’t make it easily in class, and you also get left behind with work school work.’ (P1, female, 25 years old)

Another participant said:

‘I had no concentration, and I could not study.’ (P9, female, 27 years old)

Lack of concentration, failure to cope with workload and physical discomforts negatively affected the academic progress of the pregnant student nurses. Agampondi et al. (2013:1) assert that minor maternal conditions can have major effects on the academic performance of student nurses. This is backed by Sekgobela (2008:115), who found that physical discomfort could affect student nurses’ academic performance and lead to an ongoing loss of learning time if the condition becomes severe.

Participants also reported having experienced academic stress that resulted from their failure to fulfil their responsibilities as student nurses. Kottler and Chen (2011:3) argue that stress occurs when a person is facing a situation that she or he cannot deal with. At the same time, learning and memory are adversely affected by stress (Shah & Shah 2015:12). Sharma et al. (2011:364) are of the same opinion, stating that high-stress levels do not only affect the academic performance of college students, but all aspects of the student’s health.

If pregnant student nurses can be given information about academic stress and the consequences related to it, they can learn how to reduce it. They can learn about applying coping mechanisms that may reduce complications that can lead to academic challenges. Moreover, educational institutions are obliged to protect the academic environment from stressors and should ensure that their students are not exposed to unnecessary stressful situations that may lead them to develop academic stress (Agolla & Ongori 2009:65). Students who fail to meet their academic demands may experience greater stress leading to low self-esteem, poor health habits, poor self-management choices, impaired information processing and memory disturbances (Shah & Shah 2015:12).

### Theme 2: Failure to write examinations

Some participants in this study reported that they failed to write their examinations because they either had to do catch-up programmes during the examinations, or they missed the examination dates because of illnesses or childbirth. Other reasons for not writing were that they did not have the required SANC hours because of constant absenteeism or because they missed some block tests and had low marks for their continuous assessment (CASS). One participant narrated as follows:

‘The only problem is that I did not write exam. They said I should cover the hours. I usually attended while they were writing exam.’ (P4, female, 23 years old)

Another participant said:

‘I was due to deliver during exam time [*sadness, silence*] I didn’t write.’ (P3, female, 27 years old)

According to the SANC (1985) regulation R425, as amended, a total of 4000 h of theory and practice should be completed by a student nurse before writing an examination for the particular level or training. They should have been exposed to 80% of theory and practice, failing which they are not allowed to sit for examinations (MCoN EP 2015). Some participants reported having missed lectures and tests because of being absent from class.

Absence from class means that pregnant student nurses miss lessons, block tests and assignments. They will likely fall behind in their school work, and they may be expected to ‘catch up’. At Middlesex College of Law (2011), the lecturer’s responsibilities are to remind students of the importance of regular class attendance and to update the attendance register according to the college policy. Lecturers should design methods to monitor student nurses’ attendance in large lecture rooms, and give a report to individual students about their absence (Russo-Gleicher 2015:2). This will demonstrate care on the part of the lecturer for the students who are absent often and encourage students to seek assistance from their lecturers in the form of catch-ups. However, in a study by Russo-Gleicher (2015:2), students reported that their lecturers have given up on them and failed to help them with the catch-ups, and the students thought the lecturers had no ‘caring attitude’.

Every effort should be made to ensure that pregnant student nurses do not miss writing their examinations by promoting adequate class attendance and by assisting them with catch-up programmes.

### Theme 3: Support system

Supporting students who attend college is important because students experience pressure as they try to get used to the culture of higher education (Sanacore & Palumbo 2016:25). Participant 4 said:

‘There was no support at all and I even thought of leaving school, but due to the fact that they have already called me I had to continue.’ (P4, female, 23 years old)

Attitude plays a crucial role in selecting how to behave in any given circumstances, and it impacts on an individual’s choice of action and reaction to challenges’ (Kassin et al. 2015:167). Malahlela and Chireshe (2013:141) suggest that pregnancy results in poor relationships between pregnant student nurses, their peers and lecturers, as a result of negative attitudes.

Participant 6 reported as follows:

‘Other students were pointing fingers saying ‘Ah! She’s pregnant, she doesn’t pass now and she won’t make it’.’ (P6, female, 24 years old)

Participant 8 said:

‘[*Speaking with an angry voice*] Mnn! Some of my group mates were giving me attitude because I was pregnant, they didn’t want to talk to me anymore. They said I didn’t know my purpose of being at college because I was pregnant.’ (P8, female, 23 years old)

Student nurses can help one another if they form support groups for the purpose of encouraging learning and discouraging hostility and judgemental attitudes towards pregnant student nurses. Kim et al. (2012:2) think that group participation can increase one’s ability to understand the curriculum better; hence, pregnant student nurses should belong to different groups in colleges and schools to receive assistance from various group members.

When pregnant student nurses lack a sense of belonging, they feel isolated and discriminated against.

According to Chauke (2013:37), pregnant students in her study were discriminated against during school functions, and they were found on their own during lunch breaks and other school gatherings. In a study by Gyesaw and Ankoma (2013:777), pregnant students were provoked by the other students and teachers until some of them decided to stay away from school.

Instead of getting support and encouragement, many pregnant students are stressed and embarrassed and end up not coping with their situation (Chigona & Chetty 2008:270). Malahlela and Chireshe (2013:142) say that pregnant student nurses have to deal with academic challenges and bad relations with their lecturers such that their studies become compromised. However, lecturers think that if student nurses do not report their pregnancies to them, they will not be able to offer support (Chauke 2013:67). Some lecturers think that being pregnant is an individual’s private matter and none of their business (Chigona & Chetty 2008:268). Gough and Killewald (2011:263) are of the opinion that trying to keep a pregnancy secret can affect the teachers’ and administrators’ opinions about pregnant and parenting students and also affect how the teachers and administrators will handle them.

Learning for pregnant student nurses should not be different from that of non-pregnant students.

Lecturers and ward staff should create a positive learning environment that will encourage a positive self-concept for pregnant student nurses. In a study by Rangia (2012:44), some of her participants voiced their dissatisfaction with the attitudes of their lecturers towards them that made life more difficult for them. Meanwhile, when pregnant students do not speak about problems related to their academic performance, learning becomes even more difficult, and the likelihood of achieving success is small (Sekgobela 2008:112).

Nurses have a reputation for being unapproachable and hostile towards their patients, the community and their subordinates, and they can also develop unfriendliness towards pregnant student nurses (Chigona & Chetty 2008:278). Sekgobela (2008:97) found evidence of this: 68% of her participants agreed that qualified members of staff were unfriendly to student nurses. It is also noteworthy that the remaining 32% of Sekgobela’s (2008:97) participants refused to comment on the matter.

Chigona and Chetty (2008:277) are also of the opinion that knowing how to handle pregnant students can help people understand the changes that society is presently undergoing. From the information collected from the participants, it is evident that there should be a change in attitudes towards pregnant student nurses. The classroom and ward environments should be conducive to positive interactions.

Pregnant student nurses should be encouraged to share their academic problems with their lecturers and ward staff as they depend on them for support, guidance and supervision to develop into confident and capable practitioners (Mabhuda, Potgieter & Alberts 2008:25).

Jamshidi et al. (2016:1) state that ‘student exposure to clinical learning environment is considered one of the most important factors of nursing education and it is filled with pressure and frustrations’. For pregnant student nurses to endure the pressure and frustrations of the clinical learning environment and not drop out, they have to be mentally, physically and emotionally stable (Van Den Berg & Mamhute 2013:308). Jamshidi et al. (2016:5) note that some student nurses quit the nursing profession because of problems encountered in the clinical learning areas.

Mabhuda et al. (2008:19) point to the lack of support and harassment of student nurses in the clinical learning areas as issues that negatively impact their practice.

To promote support from the clinical learning areas for pregnant student nurses, all people involved should be taught about the college pregnancy policy and other regulations that protect pregnant women.

Pregnant students should also be encouraged to make the right choice and report their pregnancy in good time. This will help ensure that they benefit from the policies and the relevant legislation. A conducive and supportive learning environment for pregnant student nurses is possible if there is support, care and good relationships between herself, colleagues, lecturers and the clinical staff (Mabhuda et al. 2008:19).

### Theme 4: Maternity leave

Pregnant student nurses and those who have given birth fail to deal with their academic duties because they have to balance the normal working hours with child- and self-care and get sufficient rest to be ready for the next day’s activities (Tanner 2012:24). The college student nurses have a dual status, being both employees and students, and they are on a personnel and salary (PERSAL) system; hence, MDOH stipulates that pregnant student nurses should be given 6 weeks of maternity leave.

During the interviews, some participants reported that they forfeited their maternity leave for various reasons. Some participants were granted maternity leave, but they came back to the college to write their examinations before their leave period ended without the permission of the clinical preceptors. Participant 7 attended the practical examinations without the knowledge of the clinical preceptor:

‘I forced myself to come and do the practical exams because I would have been left behind if I didn’t. I sacrificed my time.’ (P7, female, 28 years old)

Participant 9 said:

‘I signed for maternity leave, but then I didn’t stay for a long time at home because the exams were nearby.’ (P9, female, 27 years old)

Another participant explained:

‘After writing my examinations, I went to the clinical learning area and I asked about the maternity leave. They told me that I have forfeited the leave since I gave birth then came back to write examinations [*sadness*].’ (P3, female, 27 years old)

During puerperium, which starts immediately after the expulsion of the placenta up to 6 weeks (Marieb & Koen 2013:1021), some woman may manifest with psychological disturbances called postpartum depression. Sadock, Sadock and Ruiz (2013:839) define postpartum depression as a ‘transient mood disturbance characterized by mood change, sadness, subjective confusion and tearfulness, which may begin four to six weeks after the birth of the baby’. The depressive mood may predispose the student nurse, her colleagues and patients to danger. They may also experience problems like afterbirth pains, bleeding, exhaustion and fatigue (Agampondi et al. 2013:6).

The first month after the birth of the baby is vital for both the mother and the baby. Baker and Milligan (2008:871) state that the mother who is granted maternity leave during the first month post-delivery will be able to take care of herself and the baby and both of them can benefit from the leave. The woman will have enough time to breastfeed the baby and improve or speed up the process of involution more effectively if the woman is resting physically, emotionally and psychologically (Marieb & Koen 2013:1018).

It is important to give pregnant student nurses maternity leave to allow the body to rest and heal properly and to initialise the bond between mother and baby. Chatterji, Markowitz and Brooks-Gunn (2013:10) assert that adequate rest and permitting of longer maternity leave can be advantageous to mothers.

Bvekerwa, Choto and Shoniwa (2012:51) say that the Zimbabwean Pregnancy Policy violates the rights of pregnant women to education, work and reproduction, and they are discriminated against because of their gender. The Zimbabwean Pregnancy Policy requires that a student nurse who falls pregnant during training be given a 3-year break without pay (Bvekerwa et al. 2012:51). A survey was conducted to find out the views of the participants (pregnant student nurses from Zimbabwe) about the policy that was discriminating against them, and the results showed that pregnant student nurses did not even understand its contents (Bvekerwa et al. 2012:51). Naidoo and Kasirum (2008:13) point out the lack of maternity leave benefits for pregnant students in Nigerian colleges and universities and state that they are expelled when found to be pregnant.

The researcher is of the opinion that taking maternity leave can decrease risks leading to diseases that delay academic progress like depression (Gault et al. 2014:15).

Chatterji et al. (2013:10) also agree that susceptibility to mental conditions is greater among women who go back to work before the baby is 3 months old than among those who return to work later.

The college should promote eligibility of pregnant student nurses to maternity leave benefits.

### Limitations of the study

The findings are contextualised and could not be generalised to other colleges. Experiences of lecturers on teaching pregnant student nurses were not explored in this context; therefore, the views of lecturers cannot be conveyed.

### Implications for college and clinical setting

The interviews revealed that pregnant student nurses experienced educational challenges in both the classroom and the clinical areas. Lack of concentration and physical discomforts led to their failure to cope with their academic responsibilities and subsequent failure to progress. Failure to complete training led to decreased number of qualified professional nurses in the institution and the province.

The attitudes of other student nurses and ward staff made it impossible for pregnant student nurses to continue with training.

## Conclusion

Pregnant student nurses experienced educational challenges in both the classroom and the clinical learning areas that made them fail to cope with their studies. The examination policy is not flexible and does not allow pregnant student nurses to continue with their studies and examinations, especially when they are nearing childbirth.
